# Prevention of type 2 diabetes in British Bangladeshis: qualitative study of community, religious, and professional perspectives

**DOI:** 10.1136/bmj.a1931

**Published:** 2008-11-04

**Authors:** Clare Grace, Reha Begum, Syed Subhani, Peter Kopelman, Trisha Greenhalgh

**Affiliations:** 1 Barts and the London School of Medicine and Dentistry, Queen Mary University of London E1 2AT; 2 Public Health, Health Improvement Department, Tower Hamlets Primary Care Trust; 3 Health Advocacy Department, Tower Hamlets Primary Care Trust; 4 St George’s, University of London; 5 Department of Primary Care and Population Sciences, University of London

## Abstract

**Objective** To understand lay beliefs and attitudes, religious teachings, and professional perceptions in relation to diabetes prevention in the Bangladeshi community.

**Design** Qualitative study (focus groups and semistructured interviews).

**Setting** Tower Hamlets, a socioeconomically deprived London borough, United Kingdom.

**Participants** Bangladeshi people without diabetes (phase 1), religious leaders and Islamic scholars (phase 2), and health professionals (phase 3).

**Methods** 17 focus groups were run using purposive sampling in three sequential phases. Thematic analysis was used iteratively to achieve progressive focusing and to develop theory. To explore tensions in preliminary data fictional vignettes were created, which were discussed by participants in subsequent phases. The PEN-3 multilevel theoretical framework was used to inform data analysis and synthesis.

**Results** Most lay participants accepted the concept of diabetes prevention and were more knowledgeable than expected. Practical and structural barriers to a healthy lifestyle were commonly reported. There was a strong desire to comply with cultural norms, particularly those relating to modesty. Religious leaders provided considerable support from Islamic teachings for messages about diabetes prevention. Some clinicians incorrectly perceived Bangladeshis to be poorly informed and fatalistic, although they also expressed concerns about their own limited cultural understanding.

**Conclusion** Contrary to the views of health professionals and earlier research, poor knowledge was not the main barrier to healthy lifestyle choices. The norms and expectations of Islam offer many opportunities for supporting diabetes prevention. Interventions designed for the white population, however, need adaptation before they will be meaningful to many Bangladeshis. Religion may have an important part to play in supporting health promotion in this community. The potential for collaborative working between health educators and religious leaders should be explored further and the low cultural understanding of health professionals addressed.

## Introduction

Effective strategies for diabetes prevention in the United Kingdom must target high risk groups, including South Asians.^1^ ^2^ In the United States and Finland, intensive diet and exercise programmes have achieved a 58% reduction in incidence of diabetes in high risk groups,^3^ ^4^ but such programmes may not be directly transferable to minority ethnic communities in the UK.^5^ Lay understanding of how diabetes might be prevented has important implications for the design, delivery, and uptake of diabetes prevention programmes.^6^ Previous qualitative studies of lay understandings of diabetes in Bangladeshis have been limited to participants with established diabetes.^7^ ^8^ We explored the attitudes of British Bangladeshis without diabetes to the risk of developing diabetes and the opportunities for preventing it. We consulted religious leaders and scholars, health professionals, and community workers about diabetes prevention in the Bangladeshi community.

## Methods

This study took place in the London borough of Tower Hamlets, one of the most densely populated, multiethnic and socioeconomically deprived areas in the UK, where the age adjusted prevalence of diabetes is 5.9%.^9^ ^10^ The Bangladeshi population comprised 34% of the borough in 2001,^11^ is the largest Sylheti community outside Bangladesh^9^ with many classifying themselves as Sunni Muslims.^12^ Religion has a strong visible presence in the locality although there are dynamic sociocultural trends influencing the link between faith and identity.^13^

### Project management and governance

A project management group was set up, with representation from all participating institutions, and met about once every four months. Data were managed strictly in accordance with the data protection policy of the Queen Mary, University of London, a copy of which is available from the authors.

### Study design

The study comprised 17 focus groups in three phases: lay people (37 men, 43 women who participated in 10 groups), Islamic scholars and religious leaders (14 men and 15 women who participated in four groups), and health professionals (19 women and 1 man who participated in three groups, two men and six women in eight individual interviews). We analysed the findings from each phase and used them to design subsequent phases, allowing progressive focusing of the research.

### Phase 1: Lay people

We explored the attitudes, values, and beliefs of first and second generation Bangladeshi participants without diabetes towards the prevention of type 2 diabetes. We purposively selected participants so as to achieve maximum variation by sex, age, body mass index, and family history of diabetes. We recruited potential participants through community centres, mosques, and general practices, with groups subsequently run at the same venues. Information was available in written English or standard Bengali and an audio Sylheti version (there is no written form of Sylheti, the dialect of Bengali spoken by most London Bangladeshis).

Participants completed a short eligibility questionnaire detailing age, family history of diabetes, and generation immigrant; weight and height were measured. We invited 10 participants to each group on the basis of the purposive selection criteria. Groups were heterogeneous for most selection criteria but kept broadly homogeneous for socioeconomic status, education level, and generation of immigrant. We selected equal numbers with and without a family history of diabetes. Single sex focus groups were run by the same sex investigator (SS, RB, CG), with first generation groups run in Sylheti and second generation in English at the request of participants. To introduce different issues with probing prompt questions to explore responses in greater depth we used the topic guide (see appendix 1 on bmj.com), derived from a review of the literature, including our own previous work.

Photographs of Bangladeshi and Western meals and silhouettes of active and inactive figures were used as a basis for conversations around barriers and facilitators to food and activity choices.

After data analysis, we summarised the findings from phase 1 into vignettes using the critical fiction technique. This involves extracting key themes from the data and weaving them into a fictional story (see appendix 2 on bmj.com).^14^ These vignettes were used in the phase 2 focus groups.

### Phase 2: Islamic scholars and religious leaders

Male volunteers were recruited through mosques, Islamic forums, and Islamic schools. They held a degree in Islamic studies, and many also had higher degrees. Participating Imams suggested we also recruit leaders of female Islamic study circles. Participants completed an eligibility questionnaire giving similar details to those in phase 1 together with information on faith related training and position. We invited eight to 10 participants to each group. Vignettes based on phase 1 findings were presented to the faith leaders and their perspectives explored, focusing mainly but not exclusively on their interpretation of Islam.

### Phase 3: Health professionals

We explored the attitudes and experiences of health professionals working with the Bangladeshi community on managing weight, lifestyle, and diabetes. A focus group was run for nurses (four practice nurses, two diabetes specialist nurses, all female), dietitians (two community and four secondary care, all female), and health advocates (six women and one man). The balance between the sexes reflected the sex composition in the professional groups. Recruitment occurred through departmental, professional forums, and clinical meeting presentations as well as through individual letters of invitation. We invited seven to 10 participants to each group. Participants completed an eligibility questionnaire giving similar details to those in phase 1 and 2 plus information on any training received on lifestyle modification. Recruiting busy general practitioners to focus groups proved impossible, therefore we changed the study design to one to one semistructured interviews. We used statements and vignette style clinical scenarios to explore views. An outline topic guide is given in appendix 3 on bmj.com.

### Data processing and analysis

Focus group discussions were transcribed verbatim and where necessary translated. A sample of translations was independently checked for accuracy. The transcripts were analysed by thematic content analysis using the constant comparative method to cover identified and emerging themes.^15^ We used NVIVO, a software program for supporting qualitative data analysis. Codes are allocated by the researchers just as in manual analysis, but the software allows automated indexing, searching, and the creation of subcodes in “tree” fashion, thus allowing a large and complex dataset to be organised and analysed more easily (see www.qsrinternational.com/products_nvivo.aspx). We undertook analysis throughout the different phases so that emerging themes could be considered and incorporated into subsequent data collection (CG, TG). To enhance data synthesis and interpretation, emerging themes were discussed frequently among the team.

Data analysis was guided by the PEN-3 health promotion model (figure[Fig fig1]),^16^ which challenges and seeks to broaden the traditional westernised medical approach to disease prevention. This approach places culture at the core of the development and evaluation of successful health promotion programmes.

**Figure fig1:**
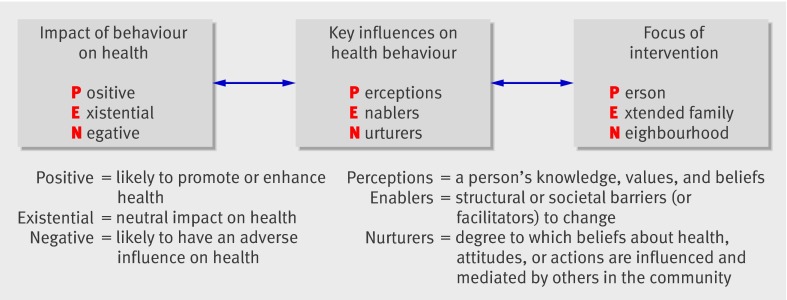
Multidimensional PEN-3 health promotion model. Adapted from Airhihenbuwa^16^

## Results

The table[Table tbl1] summarises the demographics of the focus groups in the three phases of the study. Several main themes emerged.

**Table tbl1:** Characteristics of focus groups during three phases of study

Characteristic	Phase 1	Phase 2	Phase 3
Sampling frame	Lay people without diabetes	Religious leaders and Islamic scholars	Health professionals
Total No of participants	80	29	28
Mean (SD) age (years)	35 (2)	35 (8)	41 (8)
Sex:			
Men	37	14	3
Women	43	15	25
Generation immigrant:			
First generation	62	25	Data not collected
Second generation	18	4	Data not collected
History of diabetes in first degree relative:			
Yes	30	15	Data not collected
No	50	14	Data not collected
Ethnicity:			
Bangladeshi	80	29	7
Other Asian	—	—	1
White	—	—	17
Black	—	—	3
Mean (SD) body mass index	26.4 (3.7)	Data not collected	Data not collected
Mean (SD) deprivation index	51.5 (10.1)	Data not collected	Data not collected
Study design:			
Focus groups	10	4	3
Individual interviews	—	—	8
Segmentation	Separate groups for men and women and for first and second generation immigrants	Separate groups for men and women	Separate focus groups for dietitians, nurses, advocates. All general practitioners interviewed individually by own preference

### Lay understanding of diabetes

Knowledge of diabetes was generally high and had been gleaned primarily through experience of diabetes in a relative or friend. Most participants recognised the central role of personal lifestyle choices including diet (especially sugar and fat), excess body weight, and physical inactivity in the development of diabetes. They saw the condition as at least partially preventable through lifestyle change.^17^ Some believed that kerela (traditional vegetable) and other bitter foods could prevent diabetes.

Other perceived causes of diabetes included heredity and stress, which were seen as linked to social isolation.^7^ ^8^ It was widely believed that “staying at home” played a part in poor mental wellbeing and ill health, particularly for women.

A minority of lay participants thought a family history meant diabetes was inevitable, but most thought that risk could be modified through lifestyle change. Some saw the onset of diabetes as unpredictable and its impact as cataclysmic: “We have much fear of this disease, once this disease is developed, there will be no way to save yourself” (P1/FG1/R1: female, first generation, age 49). Others, however, saw diabetes as widespread in their community and (implicitly) something not to be too concerned about.

### Living a “healthy life”

Lay participants and religious leaders both emphasised the resonance between Islamic teachings and healthy lifestyle messages such as eating a diet high in vegetables, fruit, and fish; portion control; looking after one’s body; and participating in physical activity. Rice was consistently seen as an important component of the traditional Bangladeshi diet but there was confusion over the optimum type and quantity.

Lay participants selected medium sized body images as aesthetically pleasing and associated with “good health.” Both underweight and obese body sizes were termed “weak”; such people were seen as compromised in their family and religious duties as well as predisposed to ill health. Health professionals believed (incorrectly) that Bangladeshis associate obesity with health and fertility, and hence significantly underestimated the willingness of this community to control weight.

Far from being alien to their religious identity, lay participants saw physical activity as important for mental wellbeing and a way of caring for the body, a central feature of the Muslim way of life. Physical fitness was viewed as enhancing a person’s ability to contribute to family duties, as well as a good way to control weight. Walking was seen as a valuable exercise, presenting no challenges to modesty, and was viewed by lay people and religious leaders as supported by Islamic teachings. Namaz (five times daily prayer required of devout Muslim) was widely referred to as “exercise.” Interestingly, while lay participants saw namaz as adequate exercise for maintaining health, religious leaders did not; religious leaders were in favour of conventional forms of exercise (especially walking) as part of a healthy lifestyle.

### Responsibility for diabetes prevention

Some lay participants believed that fear of the devastating impact of diabetes would motivate preventive action across the Bangladeshi community. Others, including Islamic scholars, framed prevention in a more positive but less dramatic way as part of a healthy lifestyle that all Bangladeshis should follow. When this theme was discussed, many participants asked for more specific information on what actions they should take.

“Control” was a strong theme in relation to diabetes prevention in all lay groups. People with diabetes were labelled as “out of control,” whereas those without diabetes were perceived as “in control” of food and activity choices, usually equated with having a routine or timetable. Control was generally viewed as internal (taking individual responsibility for action), with the family providing behavioural, emotional, and intellectual support. A few lay participants, however, saw control as something to be imposed and policed externally by the family:

“The family should cook the food which is advisable for him [person with or at risk of diabetes] and not to offer him any food meant for all other members of the family. They should not allow him to eat any food even though he asks for” (P1/FG5: male, first generation, age unknown)

Seeking knowledge is an important aspect of the Islamic way of life, and both lay participants and religious scholars believed that education about faith was one mechanism through which preventive messages (especially those linked to misinterpretations of religious teachings) could be conveyed. Faith was seen as linked to individuals’ confidence and motivation to change behaviour. Religious leaders were seen as trusted sources of information and support (the word Imam means teacher). They had access to large sectors of the community and were keen to incorporate messages on diabetes prevention in their teaching. They were enthusiastic about working in partnership with health professionals for mutual education and with a view to developing initiatives within the community for diabetes prevention.

### Fatalism

Many health professionals were reluctant to discuss lifestyle change in clinical consultations, partly because of their own poor cultural and religious understanding and because they perceived Bangladeshis as fatalistic (especially in relation to “the will of Allah”) and hence resistant to education on diabetes prevention: “They have to believe it to change it” (P3/FG2/R2: health advocate: female, age 35).

Interestingly, few lay participants expressed religious fatalism, but many suggested that “other people,” particularly the older generation, held such beliefs. Religious leaders saw religious fatalism as misinterpretation of Islamic teachings and were keen to address this in their role as educators.

### Social roles and expectations

Several traditional social norms were described, especially the expectation for women to remain in the home, dress modestly, and prioritise family and community over independence and social freedom—for example, not to ask someone else to mind their children. Such norms potentially conflicted with efforts to achieve health related lifestyle change. In some focus groups, women felt strong pressure to conform to traditional norms and expectations; in others (with younger and second generation women), there was support for resisting them.

The important social role of food in Bangladeshi culture was a prominent theme. Certain foods (plain rice, dhal, dry curries; one or two dishes for each meal) were considered “everyday” items and were often distinct from “special menu” foods (pilau rice, biryani, mistee; six or seven dishes for each meal) served to guests. Serving curries with reduced oil and spice content, often referred to as “white” or “pale” curries, was considered inhospitable and would be shameful to the host:

“We cook special foods in compliance of Bangladeshi society’s expectation. Otherwise people will say the new guests are not properly entertained. These foods are cooked for the fear of public scandal” (P1/FG5: female, first generation, age unknown)

Exercise in the Western sense (designated activities with special clothing, undertaken in special places such as gymnasiums) was seen as alien to the culture and identity of many first (and some second) generation Bangladeshis. Sporting exercise for women and older people was seen as inappropriate and liable to meet with the social sanction of gossip and laughter, though some thought this pressure should be ignored:

“They will say look how funny he looks, the old man has already married off his children and now he is riding bicycles and running. Even though some people have a desire to either swim or ride a bicycle they abort the idea due to fear of public scandal” (P1/FG5: male, first generation, age unknown)

“That’s the whole reason I don’t go to the gym because I have to wear trackies and all that” (P1/FG2/R4: female, second generation, age 18)

Mixed sex exercise classes were considered inappropriate for both women and men. Some saw classes for women only as acceptable, whereas others thought that these might still fail to preserve modesty or meet privacy needs. Running in public did not, in itself, meet with religious disapproval but posed challenges to modesty, particularly for women, whose bodies would be visible to men in the community. The best solution was seen as exercising at home or in a community centre, where privacy and security could be assured. But the practical challenges this posed for those living in overcrowded houses or who were unable or unwilling to travel to community centres were also highlighted. Most health professionals were uncertain about attitudes to activity in the Bangladeshi community and found this a challenging topic to explore.

### Structural and practical constraints to healthy lifestyle choices

Many Bangladeshis cited structural constraints to increasing their physical activity levels, including lack of time or money or inability to find childcare. The reluctance to travel beyond the immediate locality owing to fears about safety or difficulties with language created access problems for some first generation participants.

Practical constraints also affected dietary choices. Both male and female second generation participants reported heavy reliance on fast foods, which they saw as convenient and affordable. Traditional Bangladeshi fruits and vegetables were perceived to be expensive so were not consumed much, but first generation participants were often unfamiliar with cheaper, more readily available Western alternatives.

### Health literacy and English fluency

Lay participants identified poor fluency in English, especially in the first generation, as a major barrier to accessing and understanding basic health information. One of the consequences was a reliance on other people, often family members, to access and interpret health information on their behalf. Poor English also limited people’s willingness to travel beyond the immediate neighbourhood (owing to difficulties in reading road names or asking directions). This resulted in total reliance on local food and exercise provision.

Education was viewed by participants in all three samples as a powerful force of change by lay participants and a route to independence for women. There was also recognition that education could lead to a more liberal interpretation of religious teachings and the ability to resist traditional cultural norms:

“If the woman with hijab is intellectual and she knows the benefits of exercise, she will not care to any comment and will continue taking exercise. She will take care of her body, care for her life and not the public criticism. Then she will remember Allah’s (God’s) instructions. She will carry on doing this irrespective of what the society says” (P1/FG5/R9: female, first generation, age 40)

Many health professionals reported substantial challenges in communicating basic lifestyle information to Bangladeshis with limited health literacy, attributing this to time pressures, the difficulties of the interpreted consultation, or their own limited understanding of (and confidence to address) cultural aspects of lifestyle.

## Discussion 

We explored beliefs and values on diabetes prevention in Bangladeshis without diabetes, highlighting several positive findings. Even in a sample purposively drawn from socioeconomically deprived parts of London, lay participants knew about the link between lifestyle and diabetes, believed that lifestyle change could prevent diabetes, acknowledged individuals’ responsibility in making those changes, and viewed them as aligned with the teachings of Islam. Only a minority held strongly fatalistic beliefs that diabetes is unpredictable in onset, cataclysmic in impact, and cannot be prevented or effectively treated.

Bangladeshi religious leaders unanimously agreed that to reject the importance of self care and rely solely on Allah to protect health was a misinterpretation of Islamic teaching and must be addressed through religious education. They saw the most fruitful approach for diabetes prevention in this community to be some form of collaborative working between health educators and faith based organisations.

Our findings suggest that the main barrier to positive lifestyle change in this community is not lack of knowledge but a complex value hierarchy in which what is accepted to be healthy (small portion size, limited rich and fatty food, regular activity) is seen as less important than the social norms of hospitality, the religious requirement for modesty, and the cultural rejection of a “sporting” identity or dress (especially for women, older people, and senior members of society). Previous research has highlighted the important role of hospitality in first and second generation South Asian women (Punjabi) in Glasgow and its role in risk of heart disease.^18^

The powerful effect of social norms on an individual’s behaviour suggests that education and raising awareness alone may be insufficient to effect change in behaviour for some individuals. Our data highlighted important moral conflicts between individualist and collectivist goals (for example, the individual goal of healthy eating compared with the shame to the family of not providing guests with generous “special menu” food), and even second generation participants struggled with these conflicts. Contemporary health promotion is (arguably) built on assumptions of individualism and self investment and may need to be rethought for societies with a collectivist history.

Practical and structural barriers to a healthy lifestyle (lack of time or money, difficulties with childcare, poor housing, fear of crime, reduced access options due to limited fluency in English), were clearly evident in this study. These constraints, along with modesty and identity issues, may explain the much lower levels of participation in formal exercise programmes in Muslim communities compared with European populations, particularly for women.^19^ ^20^ ^21^ ^22^ ^23^

The strong theme among lay participants of individual responsibility and control in the prevention of diabetes conflicts with a recently published study, which reported that white respondents (with diabetes) internalised responsibility for developing diabetes through lifestyle whereas Pakistani and Indian respondents externalised responsibility, reporting general life circumstances and the migration experience as central to the cause of their diabetes.^24^ This discrepancy may be explained either by differences in ethnic subgroups, or to differences in methodology and topics explored. The differences in ethnic subgroups is supported by the sharp contrast between our own findings and those of previous studies on Bangladeshi immigrants to London,^8^ ^25^ ^26^ ^27^ ^28^ and by the fact that the London Bangladeshi community is known to have undergone substantial social transition in the past 20 years.^29^

Lay participants in our study placed high importance on family support in the prevention of diabetes. Others have shown that such support improves self management^30^ ^31^ and dietary adherence in established diabetes.^32^ ^33^ The type of support described in our study was generally positive, but social support can be problematic.^34^ ^35^ If a new behaviour is integrated with one’s own values and sense of self with no feelings of being controlled this may improve maintenance of change and can be facilitated by a sense of ownership and agency.^36^ ^37^ Family policing of healthy behaviour may convey mistrust and inhibit the process of integrated internalisation.^38^ ^39^ This theory was, however, developed in white populations and there is no empirical evidence that a more controlling form of family support is necessarily counterproductive in Bangladeshis.

Lay participants talked about the critical role that poor fluency in English played in constraining healthy lifestyle choices. Lack of basic literacy skills is recognised as an important factor in health inequalities.^40^ Our finding that “educated” Muslim women were seen as better able to resist social pressure and make up their own minds about taking exercise suggests that critical health literacy^41^ may be a prerequisite for the goal of empowerment in ethnic minority groups.

Health professionals held several incorrect views (for example, that obesity is valued in Bangladeshis^42^); they expressed a lack of confidence in their ability to provide culturally relevant advice on lifestyle. Given the increasing prevalence of overweight and diabetes and the importance of effectively addressing lifestyle related diseases it seems critical that professional education on culturally competent lifestyle interventions and approaches to delivering these must be addressed.

From a research perspective, the British Bangladeshi community has often been classed as “hard to reach,” owing to poor fluency in English and cultural barriers. We attribute the success of this study to including bilingual Bangladeshi researchers, investing time and energy in establishing trust with the community, identifying and engaging key stakeholders across the health and wider economy, and choosing a research method that capitalises on the community’s strong oral tradition.

**What is already known on this topic**

Diabetes is common in people of Bangladeshi origin living in the United Kingdom
Prevention of diabetes is possible through lifestyle change

**What this study adds**

Most Bangladeshis in this study had fair knowledge about the causes of diabetes and how to prevent it, and were keen to take personal responsibility for healthy lifestyle change
The principles of dietary and physical activity changes to prevent diabetes were seen by Bangladeshi lay people and religious leaders as aligned with the teachings of Islam, but advice on the nature of these changes must be adapted to cultural and religious norms
Health professionals admitted withholding preventive advice from Bangladeshis because of an incorrect perception of “fatalism” and lack of confidence in discussing “cultural” issues

**Key theories and sociological concepts that informed the analysis**

*Self determination theory*—extent to which the motivational basis of changing behaviour is self determined. It distinguishes between autonomous and controlled motivations. Autonomous regulation equates with personal values and choice whereas controlled regulation relates to being pressured by external forces (shame, guilt, or subjective pressure)^36^ ^37^
*Theory of reasoned action*—subjective norms (major determinants of behaviour) are the perception of social pressure to perform/not perform a behaviour based on its acceptability/appropriateness and the motivation to comply with this pressure^43^ ^44^
*Identity capital*—defined as “what individuals invest in who they are.”^45^ Tangible investment (for example, qualifications) or intangible (self esteem, critical thinking abilities, internal locus of control). These influence capacity to reflect, understand, and negotiate the challenges and opportunities met in life.^45^ Investment in the self aligns with the values of Western “individualist” societies more than with “collectivist societies whose values are oriented to duty, following shared norms, and investing in relationships with other people^45^
*Critical health literacy*—embraces not only literacy and numeracy but also the ability to think critically about advice on health and seek greater control over one’s life^17^ ^41^

## Notes

CG was funded to undertake this research through a project grant from Diabetes UK. We thank the participants who gave generously of their time; Farah Suraiya, Mohammed Lais, and Tasnuba Subhani for their translation services; and members of the project advisory group.

Ethical approval: East London and City research ethics committee.

Provenance and peer review: Not commissioned; externally peer reviewed.

## Notes

**Cite this as: ***BMJ* 2008;337:a1931
